# Damage Mechanism of PTFE/Al Reactive Charge Liner Structural Parameters on a Steel Target

**DOI:** 10.3390/ma14133701

**Published:** 2021-07-01

**Authors:** Xuepeng Zhang, Zhijun Wang, Jianping Yin, Jianya Yi, Haifu Wang

**Affiliations:** 1 School of Mechatronic Engineering, North University of China, Taiyuan 030051, China; wzj@nuc.edu.cn (Z.W.); yjp123@nuc.edu.cn (J.Y.); yjy2020@nuc.edu.cn (J.Y.); 2 State Key Laboratory of Explosion Science and Technology, Beijing Institute of Technology, Beijing 100081, China

**Keywords:** shaped charge, reactive material, reactive material jet, combined penetration explosion

## Abstract

The incorporation of reactive material damage element technology in ammunition warheads is a research hotspot in the development of conventional ammunition. The research results are of great significance and military application value to promote the development of high-efficiency damage ammunition technology. In this paper, we aimed to understand the behavior of the reactive jet and its damage effect on a steel target by undertaking theoretical analysis, numerical simulation, and experimental research. We studied the influence of structural and material parameters on the shape of the reactive jet based on autodyn-2d finite element simulation software, and the formation behavior of the reactive jet was verified using a pulsed X-ray experiment. By studying the combined damage caused by the steel target penetrating and exploding the reactive jet, the influence of the structural and performance parameters, and the explosion height of the reactive jet liner on the damage effect to the steel target was studied. A static explosion experiment was carried out, and the optimal structural and performance parameters for the reactive material and explosion height of the reactive jet liner were obtained.

## 1. Introduction

Shaped charge armor breaking projectiles are the most important warhead in active service, and are used to damage armored vehicles by metal jet, rod jet, and EFP formed by a metal charge cover under the loading of an explosive detonation wave. The penetration depth of the metal jet is deep, at up to 8–10 times that of the charge diameter, but the penetration hole diameter is small. Although it is easy to penetrate a target when it is used against light- and medium-armored vehicles, the after-effect damage depends on the kinetic energy of the subsequent jet and the debris after penetrating the target, and this effect is not always ideal. Due to the limitations of the single kinetic energy damage mechanism and mechanical penetration damage mode, metal shaped charges restrict the improvement of anti-tank shaped charge warheads’ power, and it is difficult to exert sufficient power to destroy the target in one hit, sometimes even hitting without destruction.

Fluoropolymer-based reactive materials are a new class of highly destructive energetic materials. Due to its unique performance and potentially extensive military applications, it has been highly valued by domestic and foreign ammunition and weapon research institutions and is an important frontier research direction in the field of efficient damage [1,2,3,4,5,6,7]. The main performance characteristics of fluoropolymer-based reactive materials are as follows:

(1) The reactive material generally consists of metal powders and fluorine polymer powder, prepared through mixing, molding and sinter hardening. Traditional reactive materials are highly explosive and propellant (e.g., gunpowder), and can be characterized by high strength and density, and low sensitivity. They are triggered through a traditional initiation method, such as a flame, detonator, shock wave, etc., contacting the explosive reactive material to cause a self-sustaining stable transmission [8,9,10,11,12,13,14].
(2) When a reactive material hits a high-speed collision target it goes through a process of shock loading and high plastic deformation; following this, the shear strain rate causes a fracture and the fluorine polymer decomposes, releasing fluorine with strong oxidation ability, activating the metal powder rapidly and causing an explosion/deflagration, and releasing a large amount of chemical energy, which has a significant heating effect [15,16,17,18,19,20,21,22].
(3) Compared with a traditional inert metal damage element, a reactive material can greatly enhance the structural disintegration of a target, especially due to its ability to ignite and detonate the target through the combined damage mechanism of kinetic energy penetration and an internal explosion [2,23,24,25].

These advantages of fluoropolymer-based reactive materials provide new ideas, methods and approaches for greatly improving the power of warheads [26,27]. The technical advantages of fluoropolymer-based reactive fragment materials in the application of typical warheads are as follows:

(1) The fragment killing warhead replaces the existing metal fragments, rods and shell of the warhead. By using the combined effect of penetration and internal explosion of reactive material fragments, the power of warheads can be greatly improved, especially for aerial targets such as missiles and combat aircraft, and the killing radius or K level (destruction level) can be greatly increased, and the effective strike efficiency of “one strike to destroy” can be achieved.
(2) Fluoropolymer-based reactive fragment materials can be used in penetrating warheads to replace part of the inert metal projectile body or as a projectile core. Using the combined effect of the transverse expansion and explosion of the reactive material to deal with aerial targets, such as missiles and aircraft, the K-level damage ability can be greatly improved. Attacking light armored targets, such as infantry fighting vehicles and armored personnel carriers, can significantly improve the comprehensive damage caused. Attacking oil depot/tank targets can greatly improve the ability of ignition and detonation. In addition, the use is safer and cost is lower due to the need for a fuse and explosive charge.
(3) Fluoropolymer-based reactive fragment materials can be applied to energy gathering warheads to replace the metal charge cover. The combined effect of reactive jet penetration and explosion can be used to attack light- and medium-armored targets, which can greatly improve the aftereffect of destroying armor. When attacking concrete targets, such as airport runways and bridges, reactive materials can greatly improve the damage of the internal explosion structure and is comparable to the damage efficiency of the cumulative charge-blasting two-stage series warhead.

The purpose of this paper is to clarify the formation process of the reactive jet, the combined damage effect of the penetration and explosion, and the aftereffect enhancement effect by studying the combined damage effect of the reactive jet on a steel target in order to provide a scientific basis for the application of reactive materials in warheads.

## 2. Experiments

### 2.1. Preparation of Reactive Material Liner Sample

**Figure 1:**
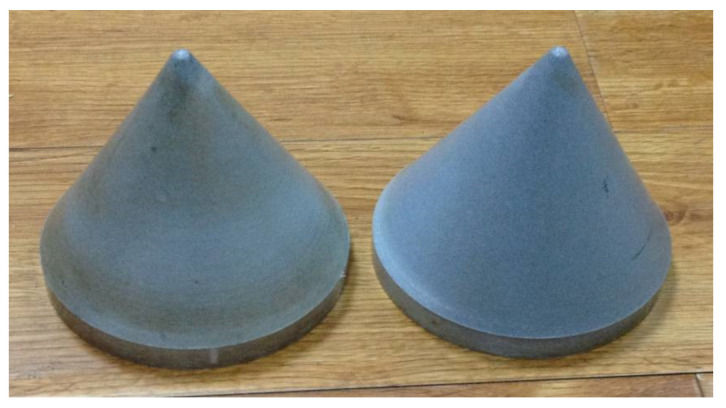
The reactive material liner.

**Figure 2:**
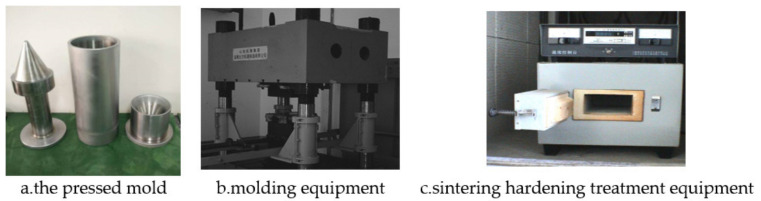
Molding equipment (**a**,**b**) and sinter hardening treatment equipment (**c**).

The reactive material liner is a kind of solid energetic damage element, prepared by adding a certain amount of energetic powder, such as metal, alloy or intermetallic compound, into the polymer powder, and then by a special molding and sinter hardening treatment. The reactive materials liner is shown in Figure 1, in which the polymer is mainly composed of a fluorine-containing polymer, such as polytetrafluoroethylene/PTFE, which is the most commonly used. PTFE (average diameter: 220 μm; DP, Guangdong, China) and Al (average diameter: 75 μm; Beijing, China) were used to fabricate the Al-PTFE sample. Reactive material projectiles were prepared by isostatically pressing powder mixtures (73.5%PTFE, 26.5%Al, by weight) encapsulated in a rigid cylindrical mandrel with a moving piston and then sintering the pressed specimens at a temperature of 375 °C in a vacuum oven. The initial powders were approximately spherical with the following average sizes: 220 μm PTFE powder, 75 um Al. And the pressed and sintered sample had a density of approximately 2.3 g/cm^3^. The preparation process of the reactive material liner is described in Figure 2.

**Figure 3:**
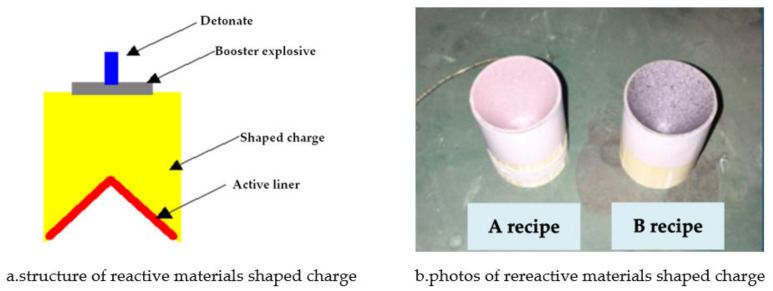
X-ray pulse test arrangement.

Considering that the matrix material is a loose, caking and fibrous powder, it needed to be crushed and screened before mixing to meet the requirements of the molding process. After the base material and filling material were mixed and dried by the mixer, they were evenly scattered into the molding cavity and scraped flat. The mold structure and photos of the real object used in the preparation are shown in Figure 2. Then, the mold was placed on the press shown in Figure 3, and the pressure was slowly and evenly increased.

In order to meet the requirements of mechanical strength, the reactive material was hardened after forming. According to the high temperature melting and cooling hardening properties of polymer matrix materials, reactive material fragments with sufficient mechanical strength can be prepared by controlling the heating and cooling processes. 

### 2.2. X-ray Pulse Test

The structure of the shaped charge of the reactive material type cover is shown in Figure 3a. It was composed of an electric detonator, a detonating charge, a powerful explosive column, and a reactive material type cover. The prepared reactive material type cover was bonded to the drug column using solid adhesive and adhesive cloth, as shown in Figure 3b.

**Figure 4:**
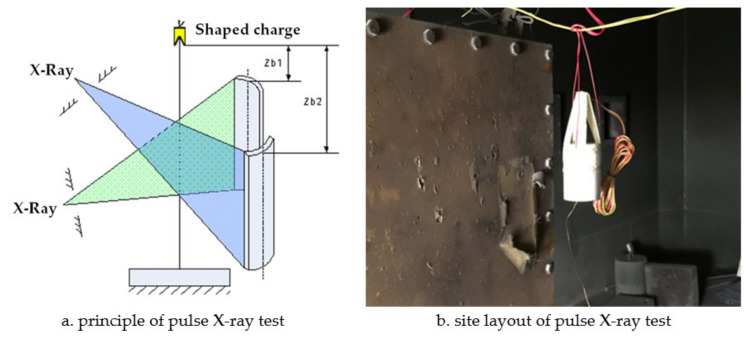
Test principle and test setup.

Pulse X-ray (model, manufacturer, city, state) technology was used to monitor the formation process of the shaped charge jet in real time to obtain the jet forming characteristics. The test principle is shown in Figure 4a and the site layout is shown in Figure 4b. The ionization field generated by the explosion of the shaped charge connected two open detonator lines, generated pulses, and triggered a flash X-ray. The first flash X-ray exposure was triggered by a 50 milliseconds delay, and the second X-ray was triggered after a delay of 80 milliseconds.

### 2.3. Penetration Test

**Figure 5:**
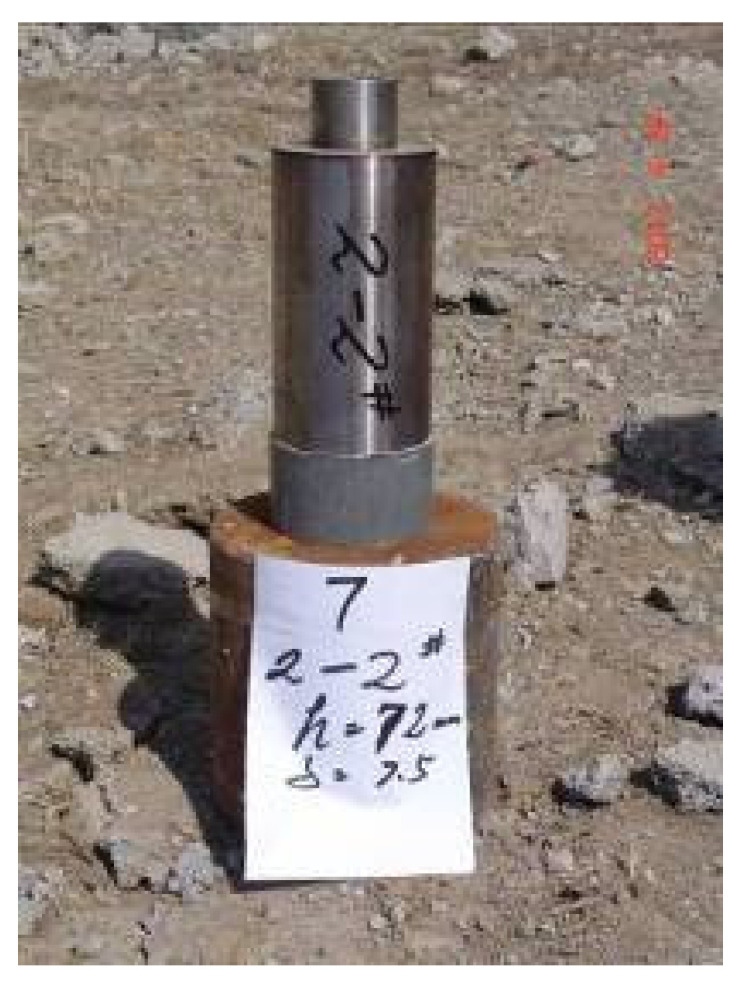
Arrangement of experimental range.

A static explosion experiment was carried out to verify the influence of the cone angle of the reactive charge on the damage effect of the reactive jet on the steel target. In the static explosion experiment, a reactive material type cover with a bottom diameter of 90 mm and density of 2.3 g/cm^3^ was used. The charge length was 180 mm and the shell was made of 45# steel with a thickness of 5 mm. The experimental principle is shown in Figure 5, which is mainly composed of a reactive charge shaped charge cover, blast height support, cylindrical steel target, and detonating device. The 45# steel cylindrical target diameter was 200 mm and its height was 200 mm.

## 3. Results and Discussion

### 3.1. Finite Element Calculation Model

The shaped charge was mainly composed of a shell, reactive liner, main charge, booster explosive, and electric detonator. The shell was made of 45# steel with a thickness of 5 mm. The main charge was 8701 explosive, with a diameter of 90 mm, length of 180 mm and density of 1.68 g/cm^3^. The material parameters and state equation of the reactive liner are shown in Table 1.

**Figure 6:**
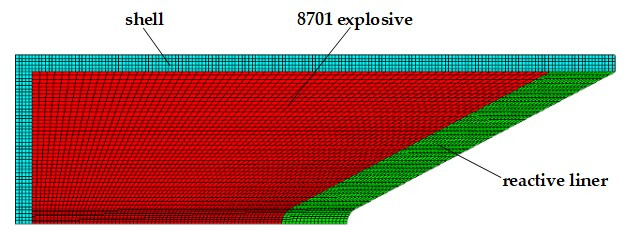
Finite element calculation model of the shaped charge.

AUTODYN-2D (ANSYS, 17.0) nonlinear dynamics simulation software was used to numerically simulate the reactive jet formation [1,28]. The formation process of the reactive jet has axisymmetric characteristics, so it can be simplified as a two-dimensional axisymmetric problem. The calculation model used the pre-processing software TureGrid for numerical modeling and meshing, and the numerical model is shown in Figure 6. The mesh distortion was significant in the formation process, so the Euler algorithm was used for the explosive and liner, while the Lagrange algorithm was used for the shell and target. The mesh size was 0.5 mm and the target was a gradient mesh. The outflow boundary was set in the Euler area and the target was a fixed constraint boundary.

**Figure 7:**
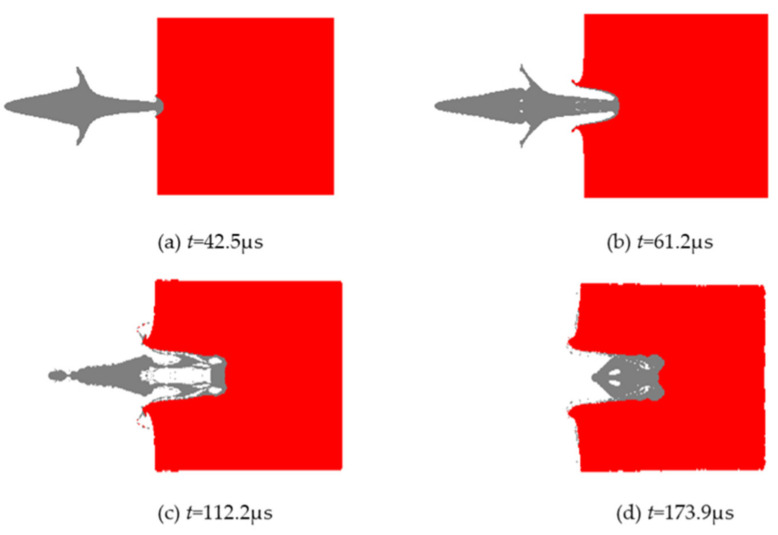
Numerical simulation results of the damage effect of the reactive jet on the steel target.

The liner formed a reactive jet under the action of detonation wave, and the numerical simulation results of the damage effect of the reactive jet on the steel target are shown in Figure 7.

Figure 7 shows that when *t* = 42.5 μs, the reactive jet begins to collide with the target, that is, this marks the beginning of the reactive jet’s effect on the steel target. At this time, atmospheric pressure is generated on the steel target and the shock wave is transmitted from the collision point to the steel target. The free surface of the steel target is cracked, and the steel target material and the jet residue are dispersed. 

When *t* = 61.2 μs, the reactive jet establishes a three-high zone in the steel target for armor breaking. During this, the quasi-steady stage, the collision pressure is small and the energy distribution of the active jet changes slowly. The parameters of armor breaking and the diameter of the hole in the steel change little, which are basically independent of the time of armor breaking. When *t* = 112.2 μs, the reactive jet appears as a cavity, the velocity reduces, and the strength of the effect on the steel target becomes significant. The subsequent reactive jet acts on the residue at the bottom of the hole, which needs to be reopened. At this time, the nail breaking speed decreases. When *t* = 173.9 μs, the velocity of the jet is very low, the jet begins to accumulate at the bottom of the invasion hole, and the break of the active jet ends.

### 3.2. Analysis of X-ray Pulse Experimental Results

**Figure 8:**
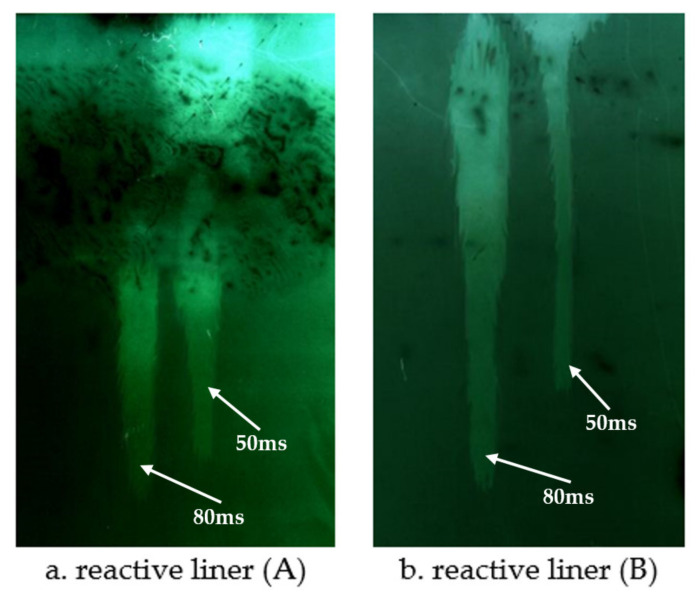
Experimental results of pulsed X-ray for reactive liner (**a**) A and (**b**) B.

Typical pulsed X-ray photos of the formation process of the reactive jet are shown in Figure 8. The pulsed X-ray experimental photos clearly distinguish the outline, head and tail shape of the reactive jet. It can be seen from the figure that the reactive charge covers different formulations and can form jets under the loading of a detonation wave, and the reactive jet has good continuity and tensile properties. The shape of the reactive jet is relatively straight and coaxiality with the detonation axis is good. At the time of images shown in Figure 8, the length of the reactive jet reached twice the diameter of the charge without shrinkage, indicating that the reactive jet has a good ductility and a large velocity gradient, that is, the jet is conducive to elongation and increased penetration capacity. It can be seen that the reactive material’s cover not only forms a stable jet, but also has good penetration ability.

**Figure 9:**
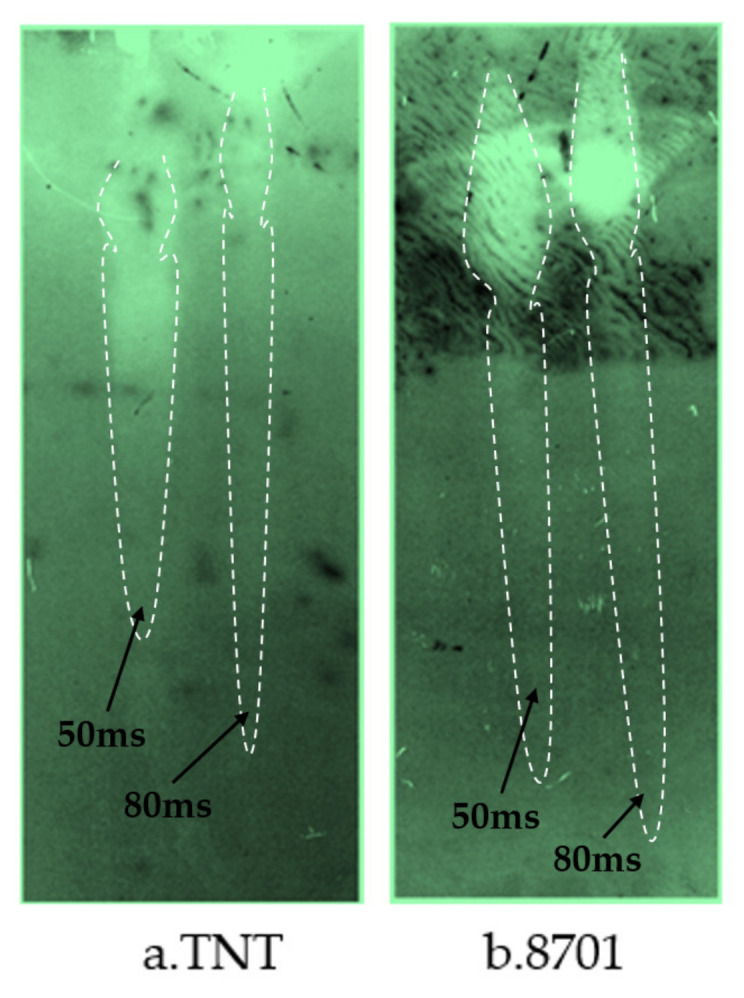
Pulse X-ray images under different charging conditions: (**a**) TNT and (**b**) 8701 explosive.

The pulse X-ray test methods and principles of reactive charge cover formation under different charge conditions were the same as mentioned above. Typical X-ray photos of reactive jet formation are shown in Figure 9. The results show that the reactive jet can be formed under different charging conditions, and the reactive shaped jet formed by 8701 explosive is thinner and the jet velocity is higher than that formed by TNT explosive.

### 3.3. Effect of Reactive Materials Type Cone Angle on Damage Effect

**Figure 10:**
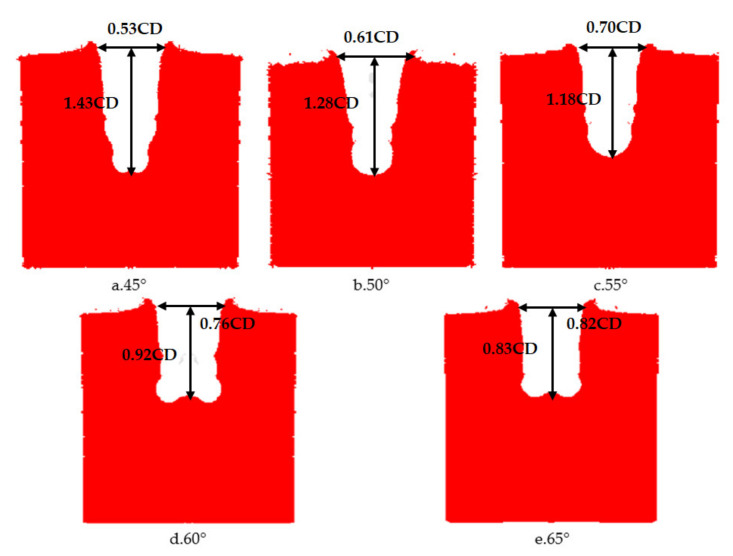
The damage effect of reactive charges wigth different cone angles on a steel target.

**Figure 11:**
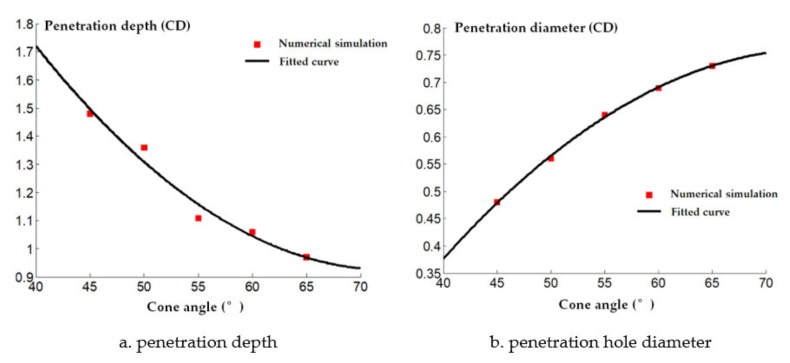
Penetration of reactive material covers with different cone angles into a steel target.

The cone angle of the shaped charge is a factor that must be considered in the design of the shaped charge structure. If the cone angle of the shaped charge is too large or small, it will have a great influence on the armor breaking effect of the reactive jet. When the cone angle is below 30°, the performance of the reactive jet is unstable. When the cone angle is between 30° and 70°, the jet has sufficient velocity and mass. When the cone angle of the charge cover exceeds 90°, the charge cover turns over in the process of deformation and EFP is formed. Therefore, it is necessary to study the influence of the change of reactive materials’ type cone angle on the penetration ability of the reactive jet in order to find the optimal cone angle. The AUTODYN–2D finite element simulation software was used to study the effect of different cone angles on the cover shaped charge’s penetrating steel target damage effect. We selected cover angles of 45°, 50°, 55°, 60° and 65°, and the cover thickness was 0.1 times the charge diameter, with a density of 2.3 g/cm^3^. The numerical simulation of the damage effect of shaped charges with different cone angles on a steel target is shown in Figure 10. The penetration depth and diameter of the shaped charge with different cone angles are shown in Figure 11.

It can be seen in Figure 11 that the jet penetration depth presents an exponential decay trend as the cone angle increases. This is because the type of shield of the cone angle is small and the detonation wave effect on the type of cover makes the crushing speed increase; thus, the reactive jet head speed increases and the jet penetration depth increases. In addition, it can be seen that the penetration diameter of the reactive jet increases as the reactive charge type cover cone angle increases. This is because the average diameter of the reactive jet formed under detonation pressure is small when the cover cone angle of the reactive charge is small.

**Figure 12:**
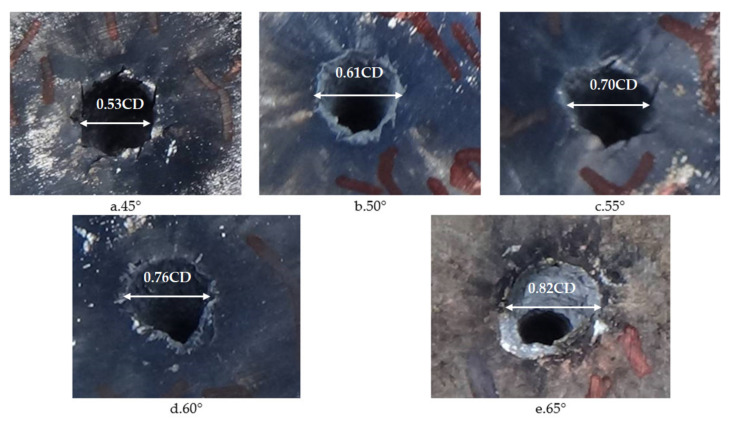
Experimental images of the shaped charge with different cone angles acting on a steel target.

Images of shaped charges with different cone angles acting on steel targets are shown in Figure 12. Figure 12 shows that the perforation of a steel target by the reactive jet is basically cylindrical, and that the difference between the upper and lower apertures is not large. The inner wall of the perforation channel is not smooth, instead showing a fish scale shape, and shear cracks appear around the perforation, with an angle of 45° with the axis, as shown in Figure 12a,c. There are residues of reactive material after the explosive reaction on the surface of the steel target, which indicates that the reactive material underwent a violent chemical reaction during penetration. The damage mechanism of the reactive material was significantly different to the metal jet. The reactive jet damaged the steel target under the combined action of kinetic energy penetration and an explosive effect. It can be seen from Table 2 that the penetration depth of the reactive jet to the steel target decreased as the cone angle of the reactive charge liner increased, which is similar to the penetration law of the metal jet, indicating that the reactive jet follows the penetration law. The penetration aperture of the reactive jet to the steel target increases with the increase of the cone angle of the reactive charge liner, which is also similar to the metal jet. In addition, it can be seen from Table 2 that the penetration depth of the reactive jet into the steel target was much smaller than the metal jet, but the penetration diameter was greater.

**Figure 13:**
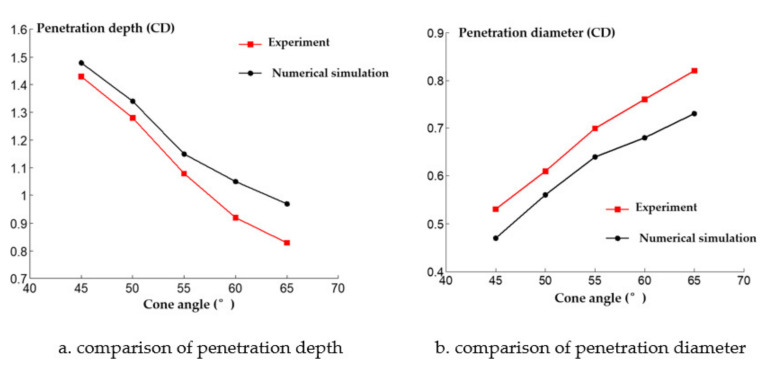
Comparison of numerical simulation and experimental results.

A comparison between the numerical simulation and experimental results of the steel target with different cone angle reactive charge liners is shown in Figure 13. It can be seen that the experimental value of the penetration depth of the steel target with different cone angle reactive charge liners is less than the numerical simulation value. This is because the explosion reaction of the reactive jet is not considered in the numerical simulation of the reactive jet penetration process. In fact, the explosive detonation wave acts on the reactive charge liner. The average pressure is about 20 GPa, which is greater than the activation pressure of the reactive material. Therefore, some reactive materials have begun to react during the formation of the reactive jet, which has a certain impact on the penetration ability of the reactive jet, and the numerical simulation value is larger than the experimental value as a result.

In addition, the numerical simulation value of the diameter of the steel target impacted by the reactive jet is smaller than the experimental value. This is because the numerical simulation does not consider the explosion reaction after the reactive jet penetration stops. In the actual experiment, the reactive jet accumulates and explodes in the hole after the penetration of the reactive jet, which has a certain enhancement effect on the radial hole expansion of the steel target, so the numerical simulation value is smaller.

Our results show that the smaller the cone angle of the reactive charge liner is, the better. However, the length of the charge is limited in the design of the structure of the shaped charge of the reactive charge liner, so there are limits to how small the cone angle can be in the reactive charge liner. Within a certain range, it is necessary to ensure the penetration ability of the reactive jet and meet the structural design of the shaped charge. The cone angle of the liner used as the research object was 55°.

### 3.4. Influence of the Reactive Charge Liner’s Wall Thickness on Damage Effect

**Figure 14:**
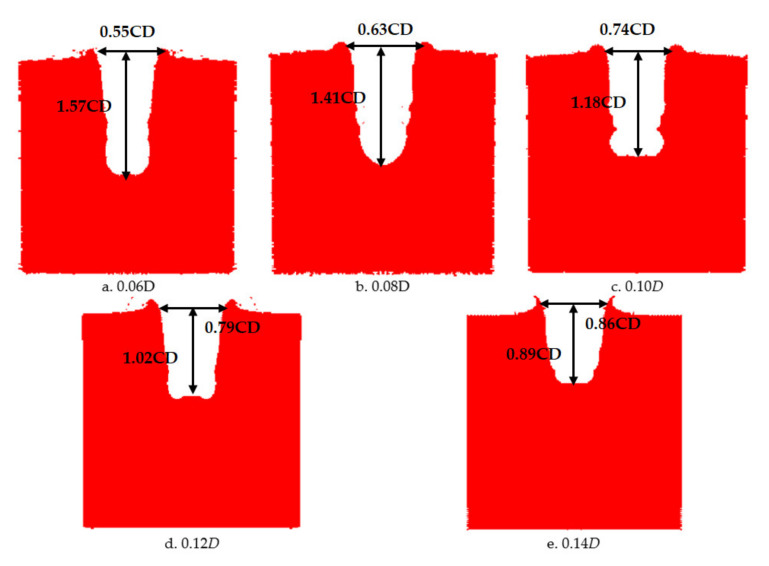
Damage effect of shaped charges with different wall thicknesses on a steel target.

**Figure 15:**
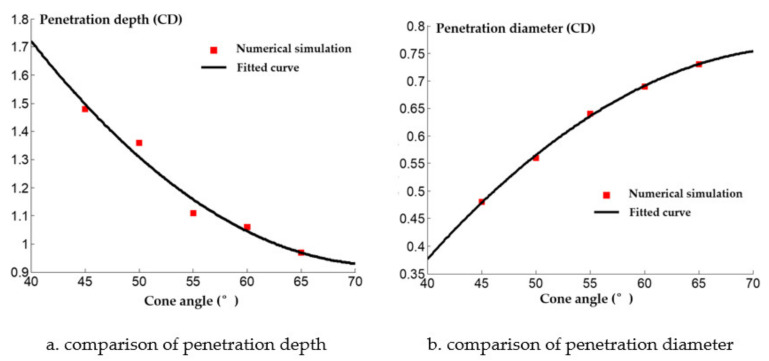
Penetration of reactive charge liners with different cone angles into a steel target.

The numerical simulation of the damage caused by the shaped charge with different wall thicknesses on a steel target is shown in Figure 14. The penetration depth and diameter of shaped charges with different wall thicknesses are shown in Figure 15.

It can be seen from Figure 15 that the penetration depth of the reactive jet into the steel target decreases as the wall thickness of the reactive charge increases. From the perspective of energy conservation, under the same charge conditions the detonation energy of the main charge is constant, but when the wall thickness of the reactive charge differs, the mass differs as well and the velocity of the reactive jet in the driving process changes. The velocity of the reactive jet increases as the wall thickness of the reactive charge increases. However, with the increase in liner thickness, the average diameter of the reactive jet also increases, resulting in an increased diameter of the reactive jet.

In order to verify the influence of the wall thickness of the reactive charge liner on the damage effect of the reactive jet on the steel target, the lowest diameter of the reactive charge liner used in the experiment was 90 mm, the density was 2.3 g/cm^3^, the cone angle was 55°, and the stand-off was one time the charge diameter. The experimental results are shown in Table 3.

**Figure 16:**
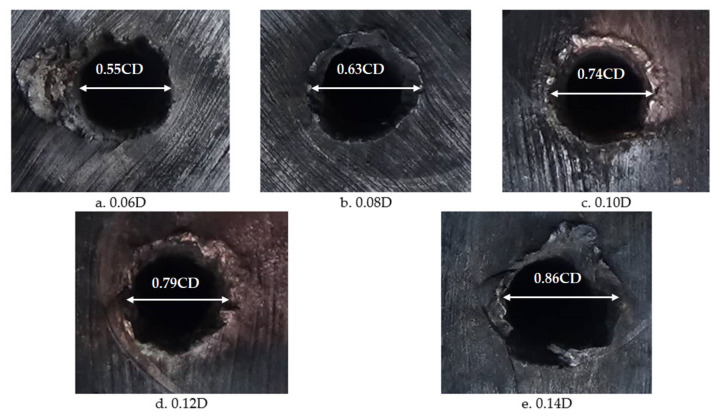
Images of shaped charges with different wall thicknesses acting on the steel target.

The images of the shaped charge with different wall thicknesses acting on the steel target are shown in Figure 16. It can be seen that there are folds around the steel target, which were caused by the reactive jet scattering the steel target material around in the pit opening stage. Furthermore, the surface texture of the steel target is no longer parallel, which indicates that, in addition to the initial aperture of the reactive jet, the reactive jet also overcame the inertial force and implosion during the penetration process, and then formed the final aperture under the combined action of these three processes, which is similar to the metal jet. The diameter of the reactive jet was much larger than the reactive jet. On the surface of the steel target, there were residues of reactive material after the explosive reaction, which indicates that the reactive material had a violent chemical reaction in the process of penetration. The damage mechanism of the reactive material was significantly different to the metal jet. The reactive jet damaged the steel target under the combined action of kinetic energy penetration and an explosive effect. It can be seen from Table 3 that the penetration depth of the reactive jet into the steel target decreased with the increase in wall thickness of the reactive charge liner, which is similar to the penetration by the metal jet. In addition, the penetration aperture of the reactive jet to the steel target increased with an increase in the wall thickness of the reactive charge liner, which is also similar to the metal jet. Therefore, the reactive and metal jets are similar, but the depth of penetration of the reactive jet into the steel target was smaller and the diameter of the penetration hole was much larger.

**Figure 17:**
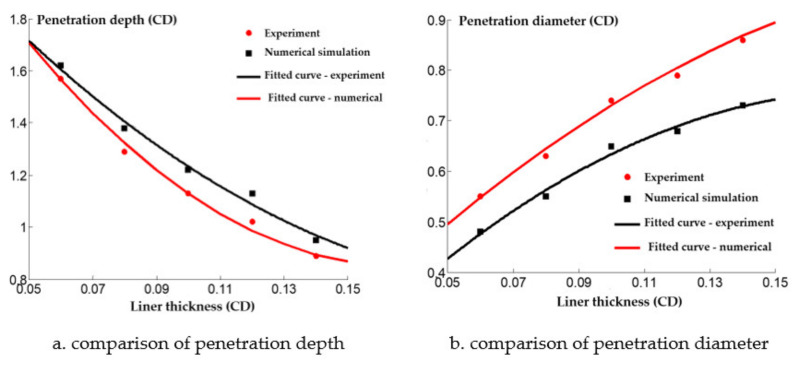
Comparison of numerical simulation and experimental results.

The comparison between the numerical simulation and experimental results of the steel target with different wall thicknesses of the reactive charge liner is shown in Figure 17. The experimental value of the penetration depth into the steel target with different wall thicknesses was consistently lower than the numerical simulation value, but the diameter of the perforation was greater than the numerical simulation value. Although there are some differences between the numerical simulation results and the experimental results, it also qualitatively shows that the penetration law of the shaped charge with different wall thicknesses is similar to the metal jet.

In summary, the smaller the wall thickness of the reactive charge liner, the better the penetration ability of the reactive jet. However, the reactive charge liner itself is an energetic liner, and the implosion reaction occurs during the penetration process. The power of the implosion mainly depends on the mass of the reactive charge liner. When the wall thickness of the reactive charge liner is too small, the mass of the reactive charge liner is small, and the explosion effect in the perforation will be weakened. In this study, the reactive charge liner with a wall thickness of 0.1 times the charge diameter was selected as the research object.

### 3.5. Influence of the Reactive Charge Liner Density on the Damage Effect

**Figure 18:**
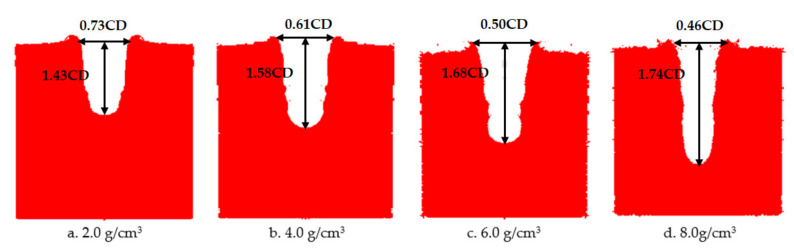
Damage effect of different reactive charge liner densities penetrating a steel target.

**Figure 19:**
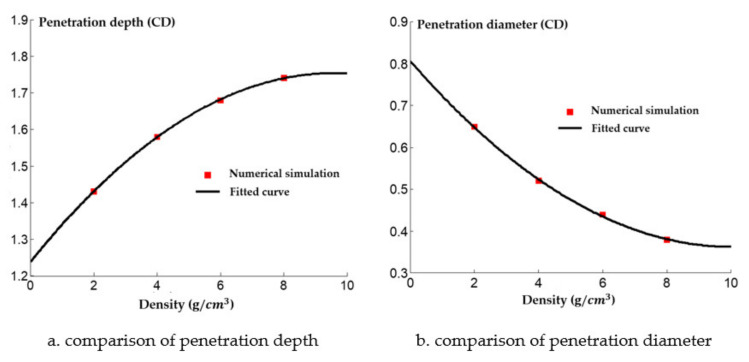
Penetration of reactive charge liners with different densities into a steel target.

The numerical simulation of the damage effect of the shaped charge with different densities on the steel target is shown in Figure 18. The penetration depth and diameter of shaped charge with different density of reactive charge are shown in Figure 19.

It can be seen from Figure 19 that the penetration depth of the reactive jet into the steel target increases with the increase of the density of the reactive charge liner. From the perspective of steady ideal hydrodynamics theory, the penetration depth of the reactive jet is not related to the velocity of the reactive jet, but is related to the length and density of the reactive jet. When the blasting height and density of the target plate remain unchanged, the armor breaking ability of the reactive jet is related to its own characteristics. The square root of the density is proportional, and the numerical simulation results are approximately consistent with the steady theory. The diameter of the reactive jet to the steel target decreases exponentially with increasing density of the reactive charge liner, which is similar to the decreasing trend of the diameter of the reactive jet with an increase in the material density in Section 2. The average diameter of the reactive jet therefore decreased, resulting in the exponential attenuation of the diameter of the reactive jet to the steel target.

In order to verify the influence of the density of the reactive charge liner on the damage effect of the reactive jet on the steel target, the smallest diameter of the reactive charge liner used in the experiment was 90 mm, the cone angle was 55° and the wall thickness was 0.1 times the charge diameter. The experimental results are listed in Table 4.

**Figure 20:**
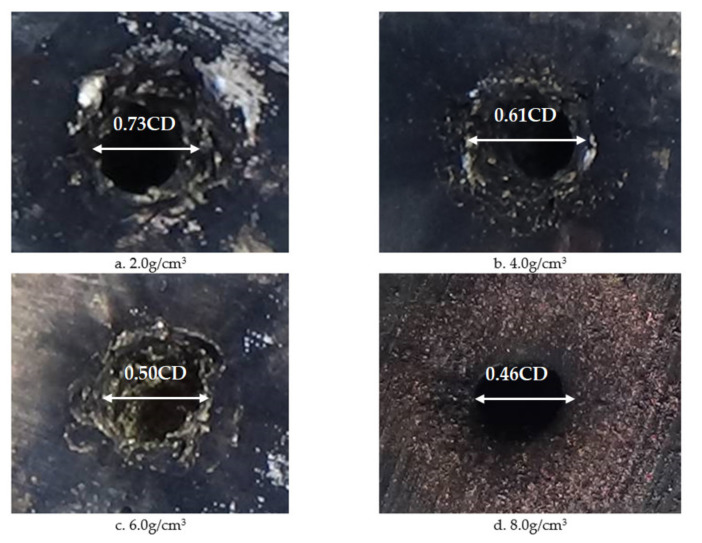
Experimental images of shaped charges with different densities acting on a steel target.

Images of shaped charges with different densities acting on the steel target are shown in Figure 20. It can be seen that as the density of the reactive charge liner increased, the penetration of the reactive jet into the steel target and the remaining unreacted metal powder on the surface of the steel target increased. This is because the increase of the density of the reactive was is due to the addition of high-density inert metal powder in the original formula. The inert metal powder did not participate in the chemical reaction of the reactive material itself but regulates the density of the reactive material.

**Figure 21:**
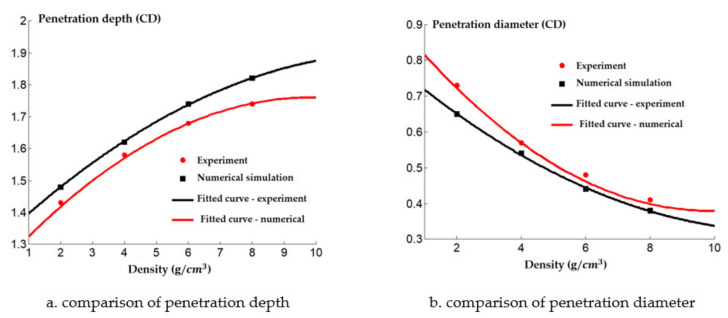
Penetration of reactive charge liners with different densities into a steel target.

A comparison between the numerical simulation and the experimental results of the steel target with different densities of reactive charge liner is shown in Figure 21. There was a certain difference between the experimental results and the numerical simulation values. The experimental results indicate that the penetration depth of the reactive jet into the steel target was less than that predicted by the numerical simulation, and the difference grows as the density increases. This is because as the density of the reactive charge liner increases, more inert metal powder is added to the reactive material, and the matching displacement is more likely to appear in the pressing process of the reactive charge liner, which has a certain impact on the penetration ability of the reactive jet. In addition, the experimental value of the penetration diameter of the reactive jet to the steel target is larger than that of the numerical simulation, and the difference becomes smaller and smaller with the increase of the density. The increase in the experimental value may have been caused by the implosion of the reactive jet, while the increase in the density of the reactive charge liner gradually reduces the energy content of the reactive jet, which leads to the weakening of the implosion effect of the reactive jet, and the difference therefore reduces.

In conclusion, the higher the density of the reactive charge liner, the better the penetration ability of the reactive jet, although the diameter of the perforation reduces, and the energy content of the reactive material decreases as the reactive charge liner density increases. This is because the increase in the density of the reactive material is due to the addition of high-density inert metal powder in the original formulation. The higher the density of the reactive charge liner, the higher the density of the added inert metal powder, which results in less energy being released by the reactive jet after the explosion reaction in the hole. Thus, it is necessary to study the mechanism of the explosion reaction. In the structural design of reactive material liners, the density of reactive material liners should not be excessive. In this paper, the density of reactive materials liners was 2.3 g/cm^3^.

## 4. Conclusions

In this paper, we studied the influence of structural and material parameters, and different blasting heights on the penetration ability of a reactive jet. The optimal structural parameters and blasting height were obtained by a static explosion experiment. The main conclusions are as follows:

(1) Based on the finite software autodyn-2d, structural parameters of the reactive charge liner were numerically simulated and our results show that the optimal cone angle of the reactive charge liner is 55°, and the optimal wall thickness of the reactive charge liner is 0.1 times the charge diameter. In addition, the influence of the performance of the reactive material and the blasting height on the penetration ability of the reactive jet was also studied, and the density of the reactive material was 2.3 g/cm^3^. The optimal explosive height was about 1.5 times the charge diameter.
(2) The numerical simulation results were verified by the static explosion experiment, and the numerical simulation results were consistent with the experimental results, which verifies the effectiveness of the numerical simulation. However, there were some differences between the numerical simulation and experimental values. The reason for the difference was that the reactive material was treated as an inert material in the numerical simulation. In fact, the reactive jet will be activated and explode during the penetration process, which has a certain impact on the penetration ability of the reactive jet.
(3) Compared with a metal jet, the penetration depth of the steel target with the reactive jet was significantly reduced, but the diameter of the steel target with the reactive jet was larger, and had a stronger burst damage effect. This shows that the reactive jet has more power to damage than a metal jet when it is used to tackle light- and medium-armored targets.

## Figures and Tables

**Table 1:** Material parameters and state equation of the reactive liner.

Materials	State Equation	Strength Model	Failure Model	Density (g/cm^3^)
Air	Ideal Gas	—	—	1.225 × 10^−3^
45# steel	Shock	Johnson–Cook	None	7.896
8701	JWL	None	None	1.680
PTFE/Al	Shock	Johnson–Cook	None	2.340
PTFE/Al	Powder Burn	Johnson–Cook	None	2.270

**Table 2:** Experimental results for reactive liner types with different cone angles (CD).

Angle (°)	Wall Thickness	Density (g/cm^3^)	Stand-Off	Target (mm)	Penetration Depth	Perforation Diameter
45	0.1	2.3	1	*Φ*200 × 200	1.43	0.53
50					1.28	0.61
55					1.18	0.70
60					0.92	0.76
65					0.83	0.82

**Table 3:** Experimental results for reactive liners with different wall thicknesses (CD).

Angle (°)	Wall Thickness	Density (g/cm^3^)	Stand-Off	Target (mm)	Penetration Depth	Perforation Diameter
55	0.06	2.3	1	*Φ*200 × 200	1.57	0.55
	0.08				1.41	0.63
	0.10				1.18	0.74
	0.12				1.02	0.79
	0.14				0.89	0.86

**Table 4:** Experimental results of steel target with different density of reactive charge liner (CD).

Angle (°)	Wall Thickness	Density (g/cm^3^)	Stand-Off	Target (mm)	Penetration Depth	Perforation Diameter
55	0.1	2.0	1	*Φ*200 × 200	1.43	0.73
		4.0			1.58	0.61
		6.0			1.68	0.50
		8.0			1.74	0.46

## Data Availability

The data is available on the request to the corresponding author.

## References

[1] Raftenberg M.N., Mock W., Kirby G.C. (2008). Modeling the Impact Deformation of Rods of a Pressed PTFE/Al Composite Mixture. Int. J. Impact Eng..

[2] Chang B.H., Yin J.P., Cui Z.Q., Liu T.X. (2015). Numerical simulation of modified low-density jet penetrating shell charge. Int. J. Simul. Model.

[3] Baker E.L., Daniels A.S., Ng K.W., Martin V.O., Orosz J.P. Barnie: A unitary demolition warhead. Proceedings of the 19th International Symposium on Ballistics.

[4] Nicolich S. (2007). Rereactive materials enhanced lethality EFP. Proceedings of the 42nd Annual Armament Systems: Gun and Missile Systems Conference and Exhibition.

[5] Daniels A.S., Baker E.L., De Fisher S.E., Ng K.W., Pham J. Bam bam: Large scale unitary demolition warheads. Proceedings of the 23rd International Symposium on Ballistics.

[6] Cai X., Zhang W., Xie W., Ni Y., Li D., Sun Y. (2015). Initiation and energy release characteristics studies on polymer bonded explosive materials under high speed impact. Mater. Des..

[7] Zhang X.F., Zhang J., Qiao L. (2013). Experimental study of the compression properties of Al/W/PTFE granular composites under elevated strain rates. Mater. Sci. Eng. A.

[8] Xiao J.G., Zhang X.P., Wang Y.Z., Xu F.Y., Wang H.F. (2016). Demolition mechanism and behavior of shaped charge with rereactive liner. Propellants Explos. Pyrotech..

[9] Xiao J.G., Zhang X.P., Guo Z.X., Wang H.F. (2018). Enhanced damage effects of multi-layered concrete target produced by rereactive materials liner. Propellants Explos. Pyrotech..

[10] Zhang X.P., Xiao J.G., Yu Q.B., Zheng Y.F., Wang H.F. (2016). Armor penetration aftereffect overpressure produced by rereactive material liner shaped charge. Acta Armamentarii.

[11] Guo H.G., Zheng Y.F., Tang L., Yu Q.B., Ge C., Wang H.F. (2019). Effect of wave shaper on rereactive materials jet formation and its penetration performance. Def. Technol..

[12] Verreault J. (2015). Analytical and numerical description of the PELE fragmentation upon impact with thin target plates. Int. J. Impact Eng..

[13] Jiang J.-W. (2012). Modeling and Simulation of JWL Equation of State for Reactive Al/PTFE Mixture. Beijing Inst. Technol..

[14] Hunt E.M., Malcolm S., Pantoya M.L., Davis F. (2009). Impact Ignition of Nano and Micron Composite Energetic Materials. Int. J. Impact Eng..

[15] Guo H.G., Zheng Y.F., Yu Q.B., Ge C., Wang H.F. (2019). Penetration behavior of rereactive liner shaped charge jet impacting steel plates. Int. J. Impact Eng..

[16] Held M. (2004). Dynamic plate thickness of era sandwiches against shaped charge jets. Propellants Explos. Pyrotech..

[17] Held M. (2007). Time distance diagram of the jet initation of covered high explosive charges. Int. J. Impact Eng..

[18] Hussain T., Liu Y., Huang F.L. (2016). Preshock desensitization phenomena during initiation of covered heterogeneous explosives by shaped charge jets. J. Energetic Mater..

[19] Mickovic D., Jaramaz S., Elek P., Miloradovic N. (2016). A model for explosive rereactive armor interaction with shaped charge jet. Propellants Explos. Pyrotech..

[20] Pyka D., Kurzawa A. (2020). Numerical and experimental studies of the Łk type shaped charge. Appl. Sci..

[21] Żochowski P., Warchoł R., Miszczak M., Nita M., Pankowski Z., Bajkowski M. (2021). Experimental and Numerical Study on the PG-7VM Warhead Performance against High-Hardness Armor Steel. Materials.

[22] Feng B., Fang X., Li C., Wang H.X., Mao S., Wu Z. (2015). An initiation phenomenon of Al-PTFE under quasi-static compression. Chem. Phys. Lett..

[23] Kobylkin I.F. (2015). Detonation initiation in shielded thin layers of explosives by shaped-charge jets. Combust. Explos. Shock Waves.

[24] Chang B.H., Yin J.P., Cui Z.Q., Liu T.X. (2016). Improved dynamic mechanical properties of modified PTFE jet penetrating charge with shell. Strength Mater..

[25] Yi J.Y., Wang Z.J., Yin J.P., Chang B.H. (2017). Numerical simulation of steel target penetration by shaped charge distended jet with a high-polymer liner. Strength Mater..

[26] Yi J.Y., Wang Z.J., Yin J.P. (2019). Simulation Study on Expansive Jet Formation Characteristics of Polymer Liner. Materials.

[27] Yi J.Y., Wang Z.J. (2019). Damage characteristics of polymer expansive jet based on the crater growth enhanced effect. Mater. Express.

[28] Smestada E., Moxnesb J.F., Degardstuena G. Modelling of Deflagration, establishing Material Data into ANSYS AUTODYN’s Powder Burn Model. Proceedings of the International Annual Conference–Fraunhofer Institute Chemical Technology.

